# Deconvolution of Transcriptional Networks in Post-Traumatic Stress Disorder Uncovers Master Regulators Driving Innate Immune System Function

**DOI:** 10.1038/s41598-017-15221-y

**Published:** 2017-11-03

**Authors:** Abolfazl Doostparast Torshizi, Kai Wang

**Affiliations:** 1 Institute for Genomic Medicine, Columbia University Medical Center, New York, NY 10032 USA; 2 Department of Biomedical Informatics, Columbia University Medical Center, New York, NY 10032 USA

## Abstract

Post-Traumatic Stress Disorder (PTSD) is a psychiatric disorder that develops in individuals experiencing a shocking incident, but the underlying disease susceptibility gene networks remain poorly understood. Breen *et al*. conducted a Weighted Gene Co-expression Network Analysis on PTSD, and identified a dysregulated innate immune module associated with PTSD development. To further identify the Master Regulators (MRs) driving the network function, here we deconvoluted the transcriptional networks on the same datasets using ARACNe (Algorithm for Reconstruction of Accurate Cellular Networks) followed by protein activity analysis. We successfully identified several MRs including SOX3, TNFAIP3, TRAFD1, POU3F3, STAT2, and PML that govern the expression of a large collection of genes. Transcription factor binding site enrichment analysis verified the binding of these MRs to their predicted targets. Notably, the sub-networks regulated by TNFAIP3, TRAFD1 and PML are involved in innate immune response, suggesting that these MRs may correlate with the innate immune module identified by Breen *et al*. These findings were replicated in an independent dataset generated on expression microarrays. In conclusion, our analysis corroborated previous findings that innate immunity may be involved in the progression of PTSD, yet also identified candidate MRs driving the disease progression in the innate immunity pathways.

## Introduction

As a debilitating neuropsychiatric disorder, Post-Traumatic Stress Disorder (PTSD) is known with several short and/or long-term symptoms such as hyperarousal, avoidance, imagery, *etc*.^1^. Unlike many other psychiatric disorders, PTSD may occur in individuals after experiencing terrifying events such as traumatic incidents, kidnapping, or natural disaster^2^. PTSD affects almost 7–8% of the general population in the United States^2^. This percentage is even higher, up to 20% in military troops being deployed in the battle fields such as Iraq or Afghanistan^3,4^. PTSD is a highly heterogeneous disorder, so that multiple types of molecular data, such as genotype, gene expression or DNA methylation^1^, may be needed to gain a deeper insight into the underlying molecular signatures contributing to the PTSD development.

Symptoms of PTSD in human subjects include emotional numbing, avoidance of traumatic events, and impaired extinction learning^5,6^. For a long time, PTSD was viewed to be a purely psychological disorder and considerable efforts were put to gain a better understanding of its symptoms in a systematic manner. Some of these psychophysiological measures are listed as follows: heart rate^7^, eye blinking (measurement of startle state), skin conductance, facial electromyogram, and cortical electroencephalographic event related potentials (ERPs, measurement of brain activity)^8,9^. PTSD has been particularly under attention from molecular point of view including gene expression and epigenetics. With regard to gene expression, a large body of studies have focused on identifying predictive and diagnostic signatures underlying PTSD development after trauma exposure. Multiple longitudinal studies have listed potential risk biomarkers^10,11^. Additionally, many other studies reported suggestive mRNA biomarkers such as FKBP5 and STAT5B^11^. Glucocorticoid activity and gene expression have long been under investigation in order to establish more effective predictive models^11–19^. Heinzlmann and Gill^20^ have compiled these studies and showed that two groups of genes including upregulated inflammatory genes and downregulated genes that participate in regulating inflammation may contribute to PTSD development. However, many of the existing approaches highlight Differentially Expressed (DE) genes and do not take into account the complex molecular interactions at the systems level in the context of gene expression networks.

Network-based methods to infer co-expression pattern of genes have been employed by several research groups. For example, groups of genes demonstrating co-expression patterns in several neuropsychiatric disorders have been indicated in a few studies^21–26^. In a recent study, Breen *et al*.^1^ have made use of the Weighted Gene Co-Expression Network Analysis (WGCNA) to elucidate co-expressed gene modules that may play certain roles in innate immunity in PTSD patients. Furthermore, PTSD blood biomarker MRs have been reported by Daskalakis *et al*.^27^ on the same data from referencere^1^ using causal upstream-regulator-analysis (URA) to predict transcription regulator activity. URA has led to 22 transcription regulators, some of which have been mentioned in reference^1^ such as STAT2. However, these studies do not probe cellular activities and the hierarchical cellular structure of the transcriptome to identify the underlying genes regulating a large blanket of downstream targets. In fact, organization of gene expression profile data into functionally meaningful information in the context of PTSD has not been addressed in the literature. This challenge, known as “reverse engineering” of cellular networks, has opened new windows to generate cellular networks in the form of graphs to overcome the common difficulties in the area of genetic networks. A number of methods have been developed for this purpose, with the most prominent one as ARACNe (algorithm for reconstruction of accurate cellular networks). ARACNe has had several successful applications in the field of cancer genomics, such as characterizing somatic mutations in cancer using network-based inference of protein activity^28^, identification of driver genes of malignant prostate cancer^29^, deconvolution of regulatory networks in human B cells^30^, construction of transcriptional network of mesenchymal transformation of brain tumors^31^, identification of causal genetic drivers in tumor^32^, and extraction of master regulators of proliferation in germinal centers^33^. ARACNe has also been recently applied in other fields, such as the identification of neurodegenerative factors^34^.

In this study, we present the successful reverse engineering of transcriptional regulatory networks to identify the Master Regulators (MRs) governing cellular processes in patients suffering from PTSD. This study is based upon ARACNe^30,35^. In essence, ARACNe initially identifies statistically significant co-regulation between gene pairs using Mutual Information (MI, i.e., an information theoretic approach) and then constructs a complete network. In the next step, indirect relationships are removed in order to address the pleiotropic associations where a target gene is regulated by more than one intermediary through applying Data Processing Inequality (DPI i.e., a well-known term in data transmission theory). The obtained final network is a robust representation of regulatory interactions or undetectable post-transcriptional modifiers in gene-expression profiles. Then, after obtaining the MRs, we analyze their activity on regulating their downstream target genes through establishing probabilistic models. Since the identified active MRs are the outcome of the expression patterns of the genes between cases and controls, therefore all of the cases and controls have been used simultaneously and MRs are shown to contribute to the development of PTSD. The entire computational results are then validated by replication experiments and literature mining. The main findings and contributions of this study are listed as follows:

- Reverse engineer the transcriptional networks from RNA-seq data based on an information theoretic approach to identify hub genes that may regulate a large blanket of downstream genes.
- Infer protein activity of hub Transcription Factors (TFs) known as MRs to discover the candidate regulatory drivers of PTSD signatures.
- Identify MRs of hub genes obtained from gene co-expression network analysis to elucidate the regulatory drivers of co-expressed modules bearing certain expression patterns.
- Validate the entire findings of this study on independent datasets along with additional experiments such as co-expression network analysis and transcription binding enrichment simulations.

## Results

### Deconvolution of Transcriptional Network of Breen *et al*. pre-deployment data

We first made use of the gene expression data generated and published by Breen *et al*.^1^. This is an RNA-seq data on peripheral blood leukocyte (PBL) that were taken from military forces being deployed in battlefields. It should be mentioned that for each sample, blood was drawn both pre- and post-deployment. In total, 47 PTSD cases and 47 PTSD-resilient control subjects were assayed with gene expression values. The raw RNA-Seq data has been processed by Breen *et al*., so we directly used the normalized expression measures. An overview of the data being used is provided in Table 1. More detail on these datasets can be found in reference^1^.

**Table 1:** An overview of the data being used.

*Time Point*	Pre-Deployment			Post-Deployment		
Dataset 1 (RNA-seq)	PTSD Cases	Controls	# Transcripts	PTSD Cases	Controls	# Transcripts
	(N = 47)	(N = 47)	22034	(N = 47)	(N = 47)	22034
Dataset 2 (microarray gene expression)	PTSD Cases	Controls	# Probe sets			
	(N = 24)	(N = 24)	12300			

The pre-deployment dataset contains expression measures on 22034 transcripts. The entire pre-deployment case and control samples were used to reverse engineer the transcription network employing ARACNe. This was done in order to reduce bias in our experiments. We reconstructed the cellular network and then pruned it to remove indirect relationships in which two genes are co-regulated through one or more intermediate entities. This allows us to observe relationships bearing significantly high probabilities of representing potential direct interactions or mediated interactions through post-transcriptional agents not being detected from gene expression profiles.

Using the P-value (a measure of the confidence of regulatory relationships between two genes) threshold of 1e-8 together with DPI = 0.1 (as recommended in^35^) leads to a repertoire of 1290844 interactions, ranging from 1 to 1772 for each individual transcript. Our goal is to focus on hub Transcription Factor (TF) genes that will be referred to as Master Regulators (MRs). We curated a list of known human TFs from three sources including FANTOM5 consortium^36^, a curated set by Vaquerizas^37^, and TRRUST^38^. A total of 2198 TFs were curated from these sources. We then focused on the MRs in the reverse engineered network and checked if any of the identified hub genes were TFs. 1903 TFs exist in the pre-deployment data. We extracted these TFs in the network along with their downstream targets (regulon). The connection degree of TFs ranges from 1 to 1396, while 12 TFs have more than 1000 targets. A complete list of the deconvoluted network of the TFs is provided in Supplementary Table 1. Directly or hierarchically, these 12 MRs account for almost 53% of the entire interactions mediated by the identified TFs.

### Protein Activity Analysis on Breen *et al*. pre-deployment data

In order to further analyze the activity of the identified MRs, we made use of the expression levels of the downstream regulon of each MR and investigated the activity degree of the identified MRs through a probabilistic algorithm called VIPER (Virtual Inference of Protein-activity by Enriched Regulon analysis^28^). VIPER infers protein activity of a MR by systematically analyzing expression of the downstream regulons that are regulated by the MRs. As previously described^28^, VIPER is based on a probabilistic framework that directly integrates target mode of regulation, that is, whether targets are activated or repressed, statistical confidence in regulator-target interactions, and target overlap between different regulators (pleiotropy) for computing the enrichment of a protein regulon in differentially expressed genes. The main advantage of VIPER compared to the other gene enrichment analysis methods such as T-profiler^39^, Fisher’s exact test^40^, and gene set enrichment analysis (GSEA)^41^ is that it uses a fully probabilistic enrichment analysis framework, supporting seamless integration of genes with different likelihoods of representing activated, repressed or undetermined targets. It also uses the probabilistic weighting of low vs. high-likelihood protein targets, while the other approaches consider the contribution of each individual gene to the signature enrichment to be binary.

**Figure 1:**
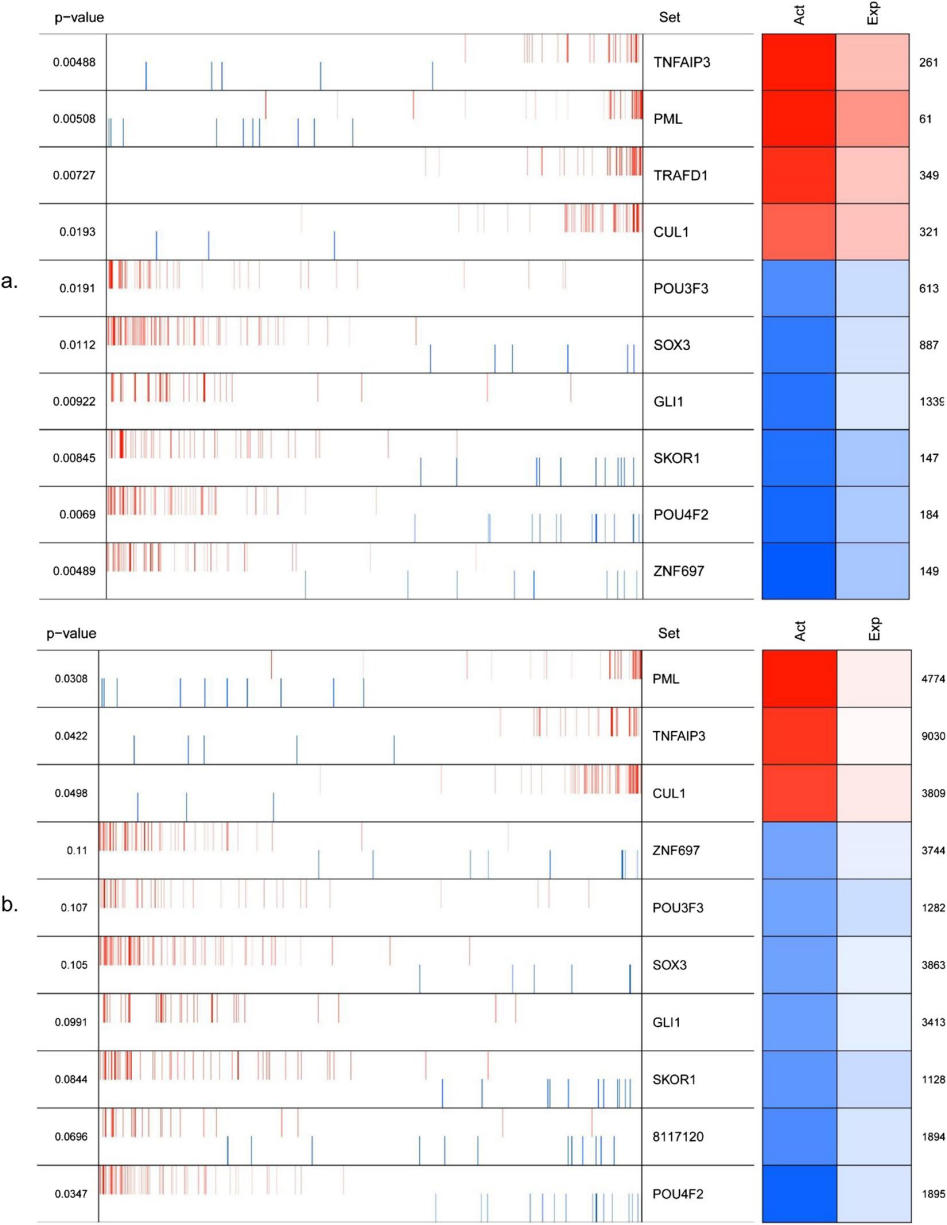
(**a**) Virtual Inference of Protein-activity by Enriched Regulon analysis. The first column on the left represents the expression of the targets of the MR where blue and red represent repression and activation, respectively. In the second column, gene symbols are represented. The Act and Exp columns represent the inferred differential protein activity and the differential expression of the identified MRs, respectively, (**b**) Representation of the enrichment of co-regulons on the gene expression signatures.

The result of VIPER analysis is summarized in Fig. 1(a), where Act and Exp columns represent the inferred differential TF activity and experimentally derived differential expression of the identified MRs, respectively. The Act and Exp generally correlate well with each other, suggesting that we reliably inferred the activity of the MRs based on expression of its target genes in the network. The activity of the MRs is inferred based on the enrichment of their regulated targets. In order to find out which genes are the enriched targets in the genetic signatures, that is, the z-score previously computed, we employed the leading-edge analysis^41^ proposed by Subramanian *et al*. to identify the genes driving the enrichment of a gene set on the signatures based on Gene Set Enrichment Analysis (GSEA). The complete list of these targets regulated by the MRs presented in Fig. 1 is provided in Supplementary Table 2. The top ten MRs provided in Fig. 1 are among the most highly connected genes identified by ARACNe.

According to previous studies^33,42^, significant activation of MRs based on their regulon analysis can cause confounding effects since many of their regulated targets might have been regulated by a bona fide activated TF. This phenomenon is called shadow effect. This is even more serious in transcriptional regulations because they are highly pleiotropic. To address this, we penalized the contribution of the pleiotropically regulated targets to the enrichment score. Since we had previously addressed the pleiotropic effects in network generation stage, we expect to observe a small number of pleiotropic connections. Overall, only 4 pleiotropic connections with weak effects were identified cementing the quality of the conducted experiments. An important point to make here is that VIPER infers the activity of MRs based on the differences of gene expression levels between cases and controls. Therefore, the implication of these MRs is that they directly impact the gene expression across the cases and controls and contribute to the transcriptional patterns that can be translated into direct impact of these MRs on PTSD development.

Furthermore, in order to predict synergistic interactions between the regulators, we computed the enrichment of co-regulons. This was defined as the intersections between regulons. Our expectation was that a gene expression signature is synergistically regulated by a combination of regulators when their corresponding co-regulons show a significantly higher enrichment on the signature than the union of the corresponding regulons^35^. We computed the enrichment of the co-regulons for the top ten regulators (Fig. 1(b)). We observed that no co-regulatory subsets of TFs are activated significantly and the same activated MRs previously observed are identified. The only extra MR found here is the ”8117120” representing the gene ID4 (the meta-data table containing gene codes is provided in Supplementary Table 3).

### Validation on Breen *et al*. post-deployment data

We repeated the same numerical experiments described above on post-deployment dataset from Breen *et al*. We used the same set up parameters in ARACNe and VIPER. The reverse engineered network constructed from the second data contained 2500722 interactions. In total, 1912 TFs were present in the network as hub genes whose connection degree ranges from 1 to 2347. The full list of the constructed network on TFs is provided in Supplementary Table 4. Feeding the constructed network into VIPER to identify MRs based on the activity of their regulons, we identified the most significant MRs. Although the overall order of the identified MR is not completely identical with the MR list of the pre-deployment data, some of the MRs among the top 30 MRs in the post-deployment data overlapped with the top 10 MRs in the pre-deployment data, including TNFAIP3, TRAFD1, POU3F3, SOX3, and STAT2. This set of MRs is thus treated as the replicated findings of this study. Additionally, PML was found to be shared among the top 20 MRs in both pre and post-deployment constructed networks.

**Figure 2:**
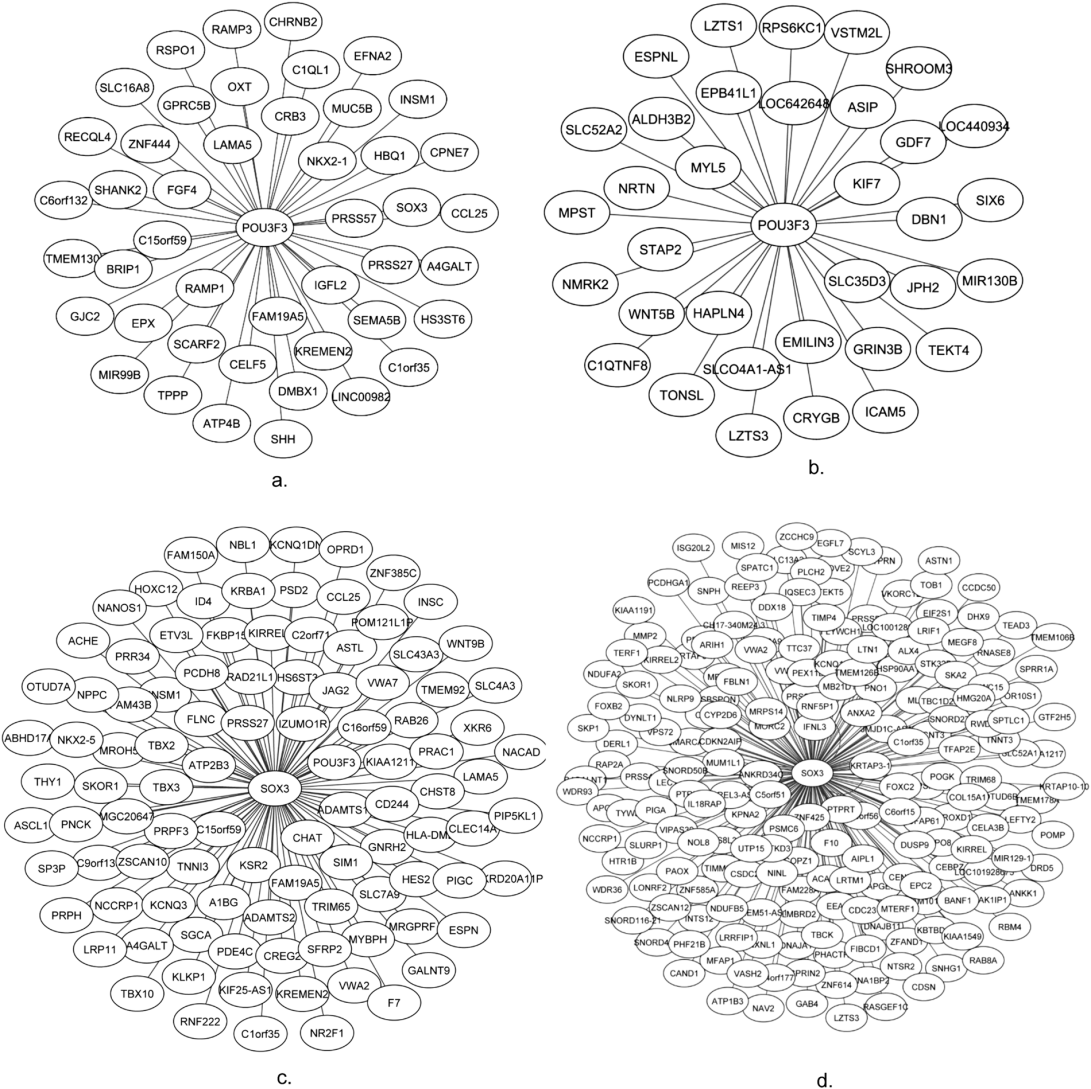
Identified regulon of the two MRs POU3F3 and SOX3 in pre and post-deployment data from Breen *et al*. (**a**) POU3F3 subnetwork in pre-deployment data; (**b**) POU3F3 subnetwork in post-deployment data; (**c**) SOX3 subnetwork in pre-deployment data; (**d**) SOX3 subnetwork in post-deployment data.

We examined the known molecular functions of the identified MRs. TNFAIP3 is associated with auto-inflammatory diseases^43^ which are driven by abnormal activation of innate immunity^44^. Similar to TNFAIP3, TRAFD1 is a regulator that controls excessive innate immune responses. The two other genes POU3F3 and SOX3 are previously reported to be involved in Central Nervous System Tuberculosis and Mental retardation, respectively. PML has been reported to be associated with leukemia^45^. It is noteworthy to mention that POU3F3 acts synergistically with SOX4 and SOX11 which are paralogs of SOX3. As an example, we generated the subnetworks of these two genes in both datasets depicted in Fig. 2. Nodes which are closer to the MR demonstrate a higher MI degree. For the both networks, there are some differences between the targets of the identified MRs though all of the common MRs are among the top active MRs. This can partially be explained by the number of samples who have been diagnosed with PTSD before combat deployment. Some of them may have not been confidently diagnosed with PTSD while they may have developed PTSD proceeding deployment, though no extra clinical information is available on individuals who actually suffered from PTSD prior to deployment.

### Further replication on the Glatt *et al*. dataset

In addition to the two aforementioned datasets, we repeated the same experiments on an independent data from Glatt *et al*.^10^. In this dataset, gene-expression levels were assayed in peripheral blood samples from 48 U.S. Marines (24 eventual PTSD cases and 24 non-PTSD comparison subjects) prior to their overseas deployment to war-zones in Iraq or Afghanistan. It has been shown that the identified panel of dysregulated biomarkers in^10^ has been significantly enriched for immune genes. We applied the same set up parameters and experimental procedures, that is, the network was reverse engineered by ARACNe and the protein activity analysis was conducted by VIPER. This dataset was totally independent of the data being used in the previous sections and are generated using microarray technology, thus has a fewer number of transcripts and genes than the RNA-Seq datasets analyzed above.

The constructed network contained 215115 interactions ranging from 1 to 494 for each gene node (Supplementary Table 5). To analyze the activity level of the hub genes to compare with the two previous experiments, this network was fed into VIPER. In this dataset, the probe sets corresponding to SOX3 and POU3F3 were not present. After analyzing the protein activity levels, we noted TRAFD1 to be among the top 15 active proteins where TNFAIP3 and PML were also observed among the top 50 active proteins, but they do not reach statistical significance after adjustment of multiple testing. An important point to note is that the data by Glass *et al*.^10^ was generated by the microarray technology with lower accuracy than RNA-Seq, and the data has approximately half the sample size as previous experiments; despite these differences, we partially replicated previously generated results, cementing the regulatory role of the identified MRs.

### Gene Ontology terms enriched by the MRs regulons

Using the targets of the identified MRs in the both datasets, we performed the Gene Ontology (GO) enrichment analysis using DAVID^46^ (all of the p-values are FDR corrected (threshold = 0.05)). The default DAVID background (whole genome background) has been used. GO terms enriched for PML regulon revealed innate immune response terms such as innate immune response (p = 1.1e-0.6), type-I interferon-mediated signaling pathway (p = 4.7e-05), immune response (p = 9.13e-10), and immune system process (p = 4.41e-07). Using the regulon of STAT2, similar immunity-related GO terms were observed including: immune system process (p = 4.9e-08), innate immune response (p = 5.7e-12), response to virus genes (p = 9.28e-11), and defense response genes (p = 3.95e-10). Fairly similar GO terms were enriched in TNFA1P3 regulon but with lower significance including: regulation of defense response to virus (p = 1.33e-01). No immunity or inflammatory-related pathways or GO terms were enriched for regulons of TRAFD1. The targets of the remaining MRs, *e*.*g*., SOX3 and POU3F3, were enriched for Dilated cardiomyopathy (P = 0.0308), Hedgehog signaling pathway (p = 0.0127), Intestinal immune network for IgA production (p = 0.0094), Axon guidance (p = 0.0389), and Vascular smooth muscle contraction (p = 0.0488). Examining the Gene Ontology (GO) terms of the regulons of SOX3 and POU3F3, we observed that many of the terms were associated with neuronal activities and development such as nervous system development (p = 0.0071), regulation of nervous system development (p = 0.001), neuron projection (P = 0.0026), and cerebral cortex neuron differentiation (p = 0.001). The same experiments were conducted for the targets of these two MRs in the post-deployment data where we observed the following pathways including: TGF-beta signaling pathway (p = 0.0094), Protein processing in endoplasmic reticulum (p = 0.0002), and Protein digestion and absorption (p = 0.0085). The GO terms being observed for these genes include riboflavin transporter activity (p = 0.0054) and protein acetous extra cellular matrix (p = 0.0058).

Our observations indicate that the identified MRs regulate downstream targets that are enriched for innate immune responses which have been reported by Breen *et al*. to be hyper-activated in PTSD, but through a different type of network analysis technique. In addition, our analysis enabled the identification of regulatory drivers of the hub genes being discovered by the WGCNA analysis, whose role in activation of innate immune responses has been thoroughly investigated.

### Transcription Factor Binding Enrichment Analysis

**Figure 3:**
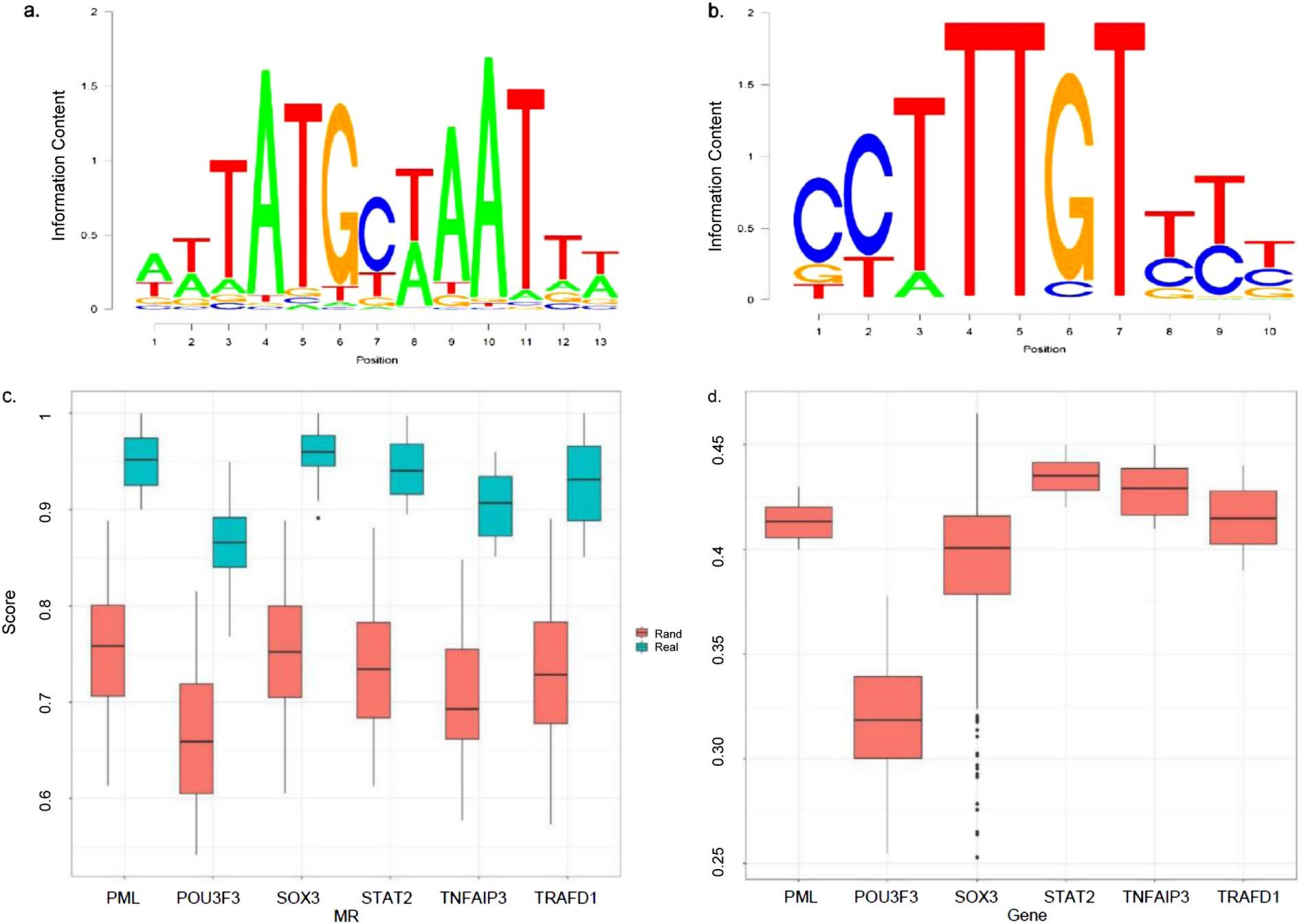
Transcription Factor Binding Enrichment Analysis. In (**a**) and (**b**) The binding motifs of POU3F3 and SOX3 are extracted from JASPAR using its available Position Weight Matrices (PWMs), respectively; (**c**) Enrichment scores are computed based on a modified Needleman-Wunsch algorithm applied by JASPAR. For each MR, a random selection of genes is compared to the observed regulons; (**d**) MI degree of the regulons of each identified MR.

To check whether binding motifs for any of the identified MRs are enriched in the promoter region of their targets (regulon), we conducted TF binding enrichment analysis (TFBEA) using JASPAR^47^. As an example, in Fig. 3, we have provided the motifs of the two MRs, POU3F3 and SOX3. We extracted the DNA sequences of the target genes 3000 base pairs upstream and 1000 base pairs downstream of their Transcription Start Sites (TSSs), then compared to the known binding motifs for these MRs. The significance threshold of 0.80 was chosen on the relative enrichment scores of the target genes. SOX3 motif sequence was enriched in its target genes with the median relative enrichment score of 0.95. As we observe in Fig. 3(c), the median MI of SOX3 target genes is almost 0.4 while the same value for POU3F3 is around 0.33. On POU3F3, the median relative enrichment score is roughly 0.87 and two genes showed enrichment scores above the 0.80 threshold. We repeated the same procedure for the rest of the MRs. To further assess the significance of the findings, for each MR, we repeated the TFBEA using random genes which were not among their targets and computed the relative enrichment scores (Fig. 3(c)). We observed that the random enrichment scores are significantly lower than the target genes of each MR, implicating a high confidence in the performed TFBEA. To gain better insights into the MI degrees of the entire target genes in the both datasets, Fig. 3(d) provides the box plots of the MI degrees for the members of each MR subnetwork.

In order to further investigate the replicated findings in both cohorts, we examined the literature for existing *in-vitro* experiments that validated the influence of the identified MRs on their target genes. Sorting the entire targets of SOX3 based on their MI, 52 genes out of the top 80 (65%) targets have been verified to be bound by SOX3 to their promoter (data from reference^48^). In comparison, 22 genes out of 80 random genes is expected to be bound by SOX3 to their promoters (27.5%). This is a strong evidence supporting the regulatory role of SOX3 on its downstream targets. Nevertheless, we caution that results generated from our network analysis are only inferences or predictions, and should be future validated in experimental systems of interest. Overall, future research on the findings of this study will open new horizons on the underlying molecular signatures of PTSD.

## Discussion

In the current study, we performed a reverse-engineering of the transcriptional network on PTSD, using the ARACNe algorithm on three gene expression datasets. We successfully deconvoluted the transcriptional networks, and identified several MRs including SOX3, TNFAIP3, TRAFD1, POU3F3 and PML that govern the expression of a large collection of downstream genes. Our analysis corroborated previous findings that innate immunity plays important roles in the progression of PTSD, yet also identified candidate MRs that drive the disease progression which may serve as potential therapeutic targets. Furthermore, we also identified POU3F3 and SOX3as potential MRs of PTSD; given their known roles in neuronal regulation, these results suggested that neuronal dysregulation may also be impaired in the development and progression of PTSD, though these findings require further validation.

Our results bear a lot of resemblance as those of Breen *et al*., despite the use of completely different statistical approaches. Using the current datasets, Breen *et al*.^1^ had conducted gene network analysis using the Weighted Gene Co-expression Network Analysis (WGCNA)^49^ to integrate expression data across thousands of genes into a higher-order system-level context to identify groups of genes within a network whose expressions are highly correlated. Using the WGCNA unsupervised method, they generated modules of co-expressed genes by combining cases and controls instead of gene-wise differential expression analysis. After replicating these experiments, they came up with two gene modules which were enriched in hemostasis wound responsiveness. Additionally, their central finding was the identification of an innate immune module associated with the development of PTSD. It is suggested that these modules are not simply the consequence of PTSD but have causal relevance to PTSD development and can in part explain pathophysiology of the disorder. The three identified gene modules in^1^ share several hub genes which are highly connected across these subnetworks including: UBE2L6, IFIH1, DTX3 L, and IFIT3, and STAT2. Although these genes contribute to the pathophysiology of PTSD, it is not clear what the drivers of these genes are and how their expression levels are regulated. The underlying regulatory machinery behind these genes will shed light not only on the mechanistic regulatory processes of these genes, but also on their co-expressed genes within the identified modules related to the activation of the immune system and inflammatory mechanisms.

One of the central findings of our study is the identification of potential MRs regulating a large body of genes which may contribute to the development of PTSD. Within these MRs and their downstream targets, we looked for the hub genes identified by WGCNA un-supervised network construction algorithm and extracted their respective MRs from our findings. These hub genes were detected as targets of PML, TRAFD1, TNFAIP3, and STAT2. The fundamental discovery is that these MRs are among the top identified MRs revealed by VIPER (Figs 1 and 2). Although STAT2 was a hub gene in one of the co-expressed modules, its regulatory role on the other co-expressed hub genes was unknown. On the other hand, the other three MRs were not captured by WGCNA. Since we have identified the potential MR of the hub genes in the WGCNA co-expression modules, we hypothesize that these MRs may modulate the expression patterns of other genes in these modules.

According to the deconvoluted network generated by ARACNe, PML, TRAFD1, TNFAIP3, and STAT2 cover 56, 44, 32, and 21 target genes, where PML and STAT2 are also connected indicating possible regulatory interactions between these TFs. They may participate in feed-forward loops having synergistic effects on each other. These five MRs were re-identified in the post-deployment reverse-engineered network as hub genes containing 1207944, and 140 targets. As noted in the previous section, TNFAIP3 and TRAFD1 are regulators that control excessive innate immune responses and STAT2 is a protein coding gene which is associated with immune response and interferon signaling pathway. This is another central finding of this work, which characterized the potential regulatory drivers of the hub genes extracted from co-expression network analysis. To gain the translational implication on how our findings can explain the role of the identified MRs, we took STAT2 as an important finding also reported in reference^1^. Looking at the pre-deployment targets of this gene, we realized that 17 out of 21 targets are DE between cases and controls indicating how such a MR can exert its effects among PTSD group. DE analysis has been performed using t-test at the threshold of P < 0.001 (FDR corrected). In fact, the identified MRs by VIPER are scored based on their exerted effects on their downstream targets in that the more distinction observed in the expression degrees of the MR targets between cases and controls leads to a higher enrichment score demonstrating the higher activity of that MR.

Our findings in this study are in line the findings from co-expression network analysis in^1^. In fact, TNFAI3, TRAFD1, and STAT2 are regulators to control excessive innate immune responses. Our results show that these genes are among the major regulators of the innate immune response module discovered by WGCNA analysis. On the other hand, POU3F3 and SOX3 have not previously been reported as MRs for PTSD. Looking at the targets of these two genes, we observed that many of these target genes are hubs themselves covering a large number of genes. This is an implication of hierarchical structure of the constructed networks. Many of the well-known PTSD biomarkers such as FKBP5, STAT5A, HOXD3, VWA3, ALDH3B2, *etc*. were covered directly or as the secondary neighbors of the identified MRs. We were able to capture OAS2 as one of the most influential genes as the markers of the antiviral interferon response that was associated with an increased risk of PTSD^50^. This gene was among the targets of PML (MI = 0.38) and STAT2 (MI = 0.36), and our finding partially explains the genetic roots controlling its regulation. A fundamental question raised by Breen *et al*.^1^ on the constructed co-expressed gene modules was that how innate immunity genes are over-expressed prior to trauma, and this question may be partially answered in our study. We hypothesize that the activation/deactivation of STAT2 can partially explain over/under expression of its targets which are mainly enriched in innate immunity pathways. This is due to the fact that STAT2 is triggered by stress^19^. As a result of its expression, it can affect its targets leading to enrichment of the innate immune pathways. One possibility is that the targets of STAT2 are not necessarily regulated because of foreign stimuli, but they are regulated only because of the activation of their respective MR. In addition, we were able to capture many of the known PTSD markers such as FKBP5 and STAT5B that were not captured by the WGCNA.

Using the brain transcriptome profiles of a mouse model simulating PTSD in stress-related brain regions including amygdala and hippocampus^51^, we analyzed the expression patterns of the identified MRs in amygdala and hippocampus. We noted the following genes to be differentially expressed (FDR corrected) including: SOX3 (p = 0.0065), TNFAIP3 (p = 0.0428), TRAFD1 (p = 0.0431), POU3F3 (p = 0.026), and STAT2 (0 = 0.0139). PML was not found to be differentially expressed in amygdala. This data can be accessed in GEO under accession number GSE45035.

For each identified MR, we also mined the literature to check whether or not they are expressed in stress-related regions in the brain such as amygdala, hippocampus etc. A recent study demonstrates STAT2 to be differentially expressed in a well-characterized rat model of temporal lobe epilepsy evoked by electrical stimulation of the amygdala^52^. SOX3 expression in neonatal and adult mouse brains suggests potential involvement of this gene in regulating persistent neural stem cells and neurogenesis^53^. Such a finding confirms persistent SOX3 expression in mature neuronal populations, suggesting further roles of this gene in neuronal function in stress-related regions of the brain. As a key transcription factor involved in cortical development^54^, POU3F3 has been verified to be overexpressed in different brain regions and may play role in the development of the nervous system^55^. Despite lack of evidences on expression of PML in brain, recent studies have found PML in neurons and as well as its functions in many aspects of the nervous system such as brain development, plasticity, and response to proteins causing neurodegenerative disorders^56^.

In addition, we checked the expression of the MRs in the Genotype-Tissue Expression (GTEx) Project portal to see if our genes of interest are expressed in stress-related regions of the brain. Supplementary Figures 1–5 demonstrate the expression patterns of SOX3 and POU3F3 across different human tissues based on RNA-seq experiment results. The expression patterns of these two MRs across brain regions are evidently higher than other tissues but a similar pattern was not observed on the rest of the identified MRs, indicating that they are not highly expressed in brain tissues.

In order to analyze possible overlap between our findings in PTSD and other neuropsychiatric disorders, we re-did the computations on two other diseases including Autism Spectrum Disorder (ASD) and Schizophrenia (SCZ). We ran the whole pipeline on a gene expression data generated from Autism Spectrum Disorder (ASD) patients^57^. Sorting the highly active proteins based on VIPER results, none of the identified PTSD MRs were observed among the top 100 ASD findings. Additionally, we ran the whole pipeline on the largest schizophrenia (SCZ) RNA-seq data to date^58^. Similar to ASD, none of the PTSD MRs were observed among the top 100 SCZ MRs. Therefore, our findings appear to be specific to PTSD, but may still be extended to other neuropsychiatric disorders when tissue-specific large-scale gene expression data sets become available.

Our study has a number of limitations. This study was based on gene expression data from peripheral blood. Although our findings have been replicated in several different data cohorts, further investigation is necessary to better understand how neuronal activities contribute to PTSD. This is due to the fact that gene expression patterns are highly tissue-specific and gene expression measures in blood may not totally reflect the regulatory machinery in brain or other related tissues. Additionally, the need for large sample size is also an important requirement in the area of gene expression network analysis. We believe that larger cohorts may reveal PTSD signatures that may not be easily captured when the sample size is small. Additional improvements to the current network analysis can be the application of latent biological knowledge such as protein-protein interactions or biological pathways during the course of network reverse-engineering process, which may improve the power to uncover sophisticated gene-gene interactions and regulatory roles of MRs. Finally, we should state that these findings are only based upon computational inferences, so that conducting *in vivo* experiments on predictions made in this study can be of paramount importance for future investigations.

## Methods

### Study Cohort

We made use of the gene expression data generated and published by Breen *et al*.^1^ and Glatt *et al*.^10^. Breen *et al*. used RNA-seq to assay gene expression on peripheral blood leukocyte (PBL) that were taken from two independent groups of military forces being deployed in battlefields. It should be mentioned that for each sample, blood was drawn both pre-and post-deployment. Further information can be found in^1^. For validation, we also analyzed an independently generated gene expression dataset from a separate, non-overlapping group of 50 U.S. Marine participants (Glatt *et al*.^10^). Blood samples were treated in an identical fashion as described above; however, the gene expression was measured on the Affymetrix Hu-Gene 1.0 ST Array. These datasets are publicly available in Gene Expression Omnibus (GEO) under the accession number GSE64814.

### ARACNe network reconstruction

ARACNe (Algorithm for the Reconstruction of Accurate Cellular Networks)^35^, an information-theoretic algorithm for inferring transcriptional interactions, was used to identify candidate transcriptional regulators of the transcripts annotated to genes. First, mutual interaction between a candidate TF($x$) and its potential target ($y$) was computed by pairwise mutual information, $MI(x,y)$, using a Gaussian kernel estimator. MI was thresholded based on the null-hypothesis of statistical independence (P < 0.05, Bonferroni corrected for the number of tested pairs). Other key elements such as kernel width of the estimator can be set manually or automatically in the code (we used the recommended automated option). Second, the constructed network was trimmed by removing indirect interactions by data processing inequality (DPI), a property of the MI. By this, pleiotropic associations will be removed. Therefore, for each $(x,y)$ pair, a path through another $TF(z)$ was considered and every path pertaining the following constraint were removed $MI(x,y) < \,min(MI(x,z),MI(z,y))$. In fact, ARACNE eliminates the statistical dependencies which can be of an indirect nature e.g., two genes which are segregated by an intermediate step in a transcriptional cascade. These genes usually have correlated expression patterns that can lead to high MIs and might be selected as MRs. After some post-processing steps, the final output of ARACNe is the adjacency matrix of the constructed network that can be used for further analysis such as evaluation of protein activities. ARACNe software is available and can be downloaded from reference^35^.

### VIPER analysis of regulon enrichment

The enrichment of regulons on gene expression signatures for each MR was tested by the VIPER algorithm^28^. In VIPER, first, the gene expression signature is obtained by comparing two groups of samples representing distinctive phenotypes or treatments, that is, those with and without PTSD. To generate a quantitative measurement of difference between the groups, Students t-test is used. As an alternative, single sample-based gene expression signatures can be obtained by comparing the expression levels of each feature in each sample against a set of reference samples by any suitable method, including for example Students t-test, Z-score transformation or fold change; or relative to the average expression level across all samples when clear reference samples are not available. In the next step, regulon enrichment on the gene expression signature is computed using Analytic rank-based enrichment analysis (aREA). At the end, significance values (P-value and normalized enrichment score) were computed by comparing each regulon enrichment score to a null model generated by randomly and uniformly permuting the samples 1000 times. As an internal function in VIPER, aREA tests for a global shift in the positions of each regulon genes when projected on the rank-sorted gene expression signature. Following up on the workin^59,60^, the mean of the quantile-transformed rank positions as test statistic (enrichment score) are used. The enrichment score is computed twice: first by a one-tail approach, based on the absolute value of the gene expression signature (i.e., genes are rank-sorted from the less invariant between groups to the most differentially expressed, regardless of the direction of change); and then by a two-tail approach, where the positions of the genes whose expression is repressed by the regulator are inverted in the gene expression signature before computing the enrichment score. The one-tail and two-tail enrichment score estimates are integrated while weighting their contribution based on the estimated mode of regulation through a procedure we call three-tail approach. The contribution of each target gene from a given regulon to the enrichment score is also weighted based on the regulator-target gene interaction confidence. At the end, the statistical significance of the enrichment scores are estimated by comparison to a null model generated by permuting the samples uniformly at random. The final outcome of the VIPER is a list of MRs based on their enrichment scores along with their respective targets. VIPER software package is available on Bioconductor at http://bioconductor.org/packages/viper/.

### Transcription factor binding site enrichment analysis

Human reference genome (version GRCh37.p13) was used to extract the DNA sequence around transcript start sites(TSSs) for transcription binding enrichment analysis. We obtained the gene coordinates from Ensembl BioMart tool^61^ and scanned 3000 upstream and 1000 downstream of the TSS. The motifs of the TFs were obtained from JASPAR and the extracted sequences of each target were then fed into JASPAR and analyzed versus their corresponding TFs. JASPAR database contains Position Weight Matrices (PWM) for each TF. These matrices have four rows representing A, C, G, and T bases and each column represents the relative weight of that pair in that position obtained from the frequency of that base in that position. Then, using the PWM of the TF, JASPAR employs the modified Needleman-Wunsch algorithm to align the motif sequence with the target sequence in that the input sequence is scanned to check whether or not the motif is enriched. The output is the enrichment score of the input TF in the designated target genes.

## Electronic supplementary material

Supplementary File

Supplementary Table 1

Supplementary Table 2

Supplementary Table 3

Supplementary Table 4

Supplementary Table 5
