# Supplementary material for: Deconvolution of Transcriptional Networks in Post-Traumatic Stress Disorder Uncovers Master Regulators Driving Innate Immune System Function
Source: Sci Rep. 2017 Nov 3;7:14486. doi: 10.1038/s41598-017-15221-y (PMC5670244; doi:10.1038/s41598-017-15221-y)
Supplement: Supplementary file 1 — Supplementary File [file 41598_2017_15221_MOESM1_ESM.pdf]

Deconvolution of Transcriptional Networks in Post-Traumatic Stress Disorder Uncovers Master Regulators Driving Innate Immune System Function

Abolfazl Doostparast Torshizi[1], Kai Wang[1,2*]

[1] Institute for Genomic Medicine, Columbia University Medical Center, New York, NY 10032, USA.

[2] Department of Biomedical Informatics, Columbia University Medical Center, New York, NY 10032, USA.

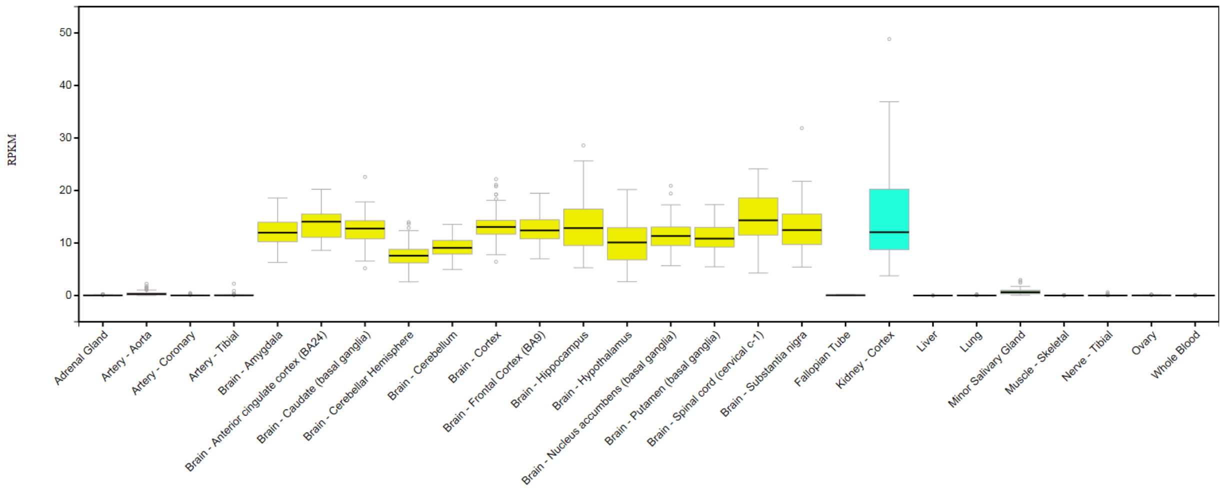

SFigure 1. POU3F3 expression across different tissues in GTEx

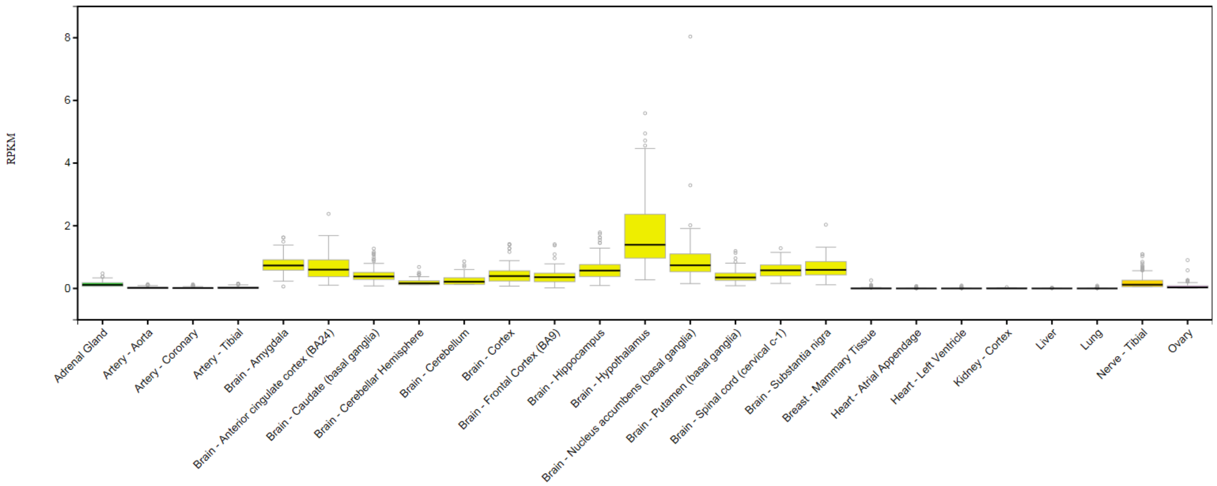

SFigure 2. SOX3 expression across different tissues in GTEx

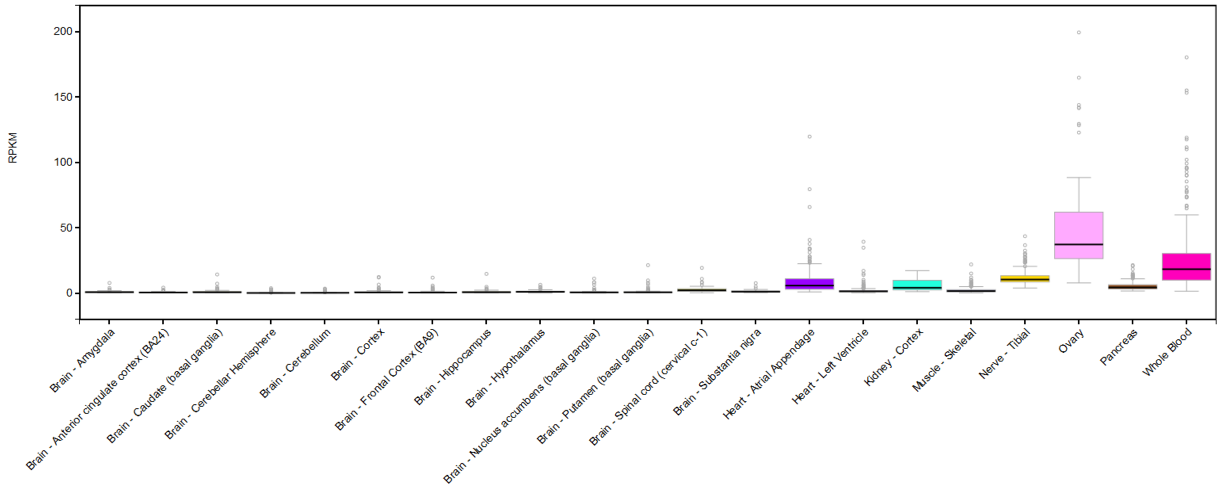

SFigure 3. TNFAIP3 expression across different tissues in GTEx

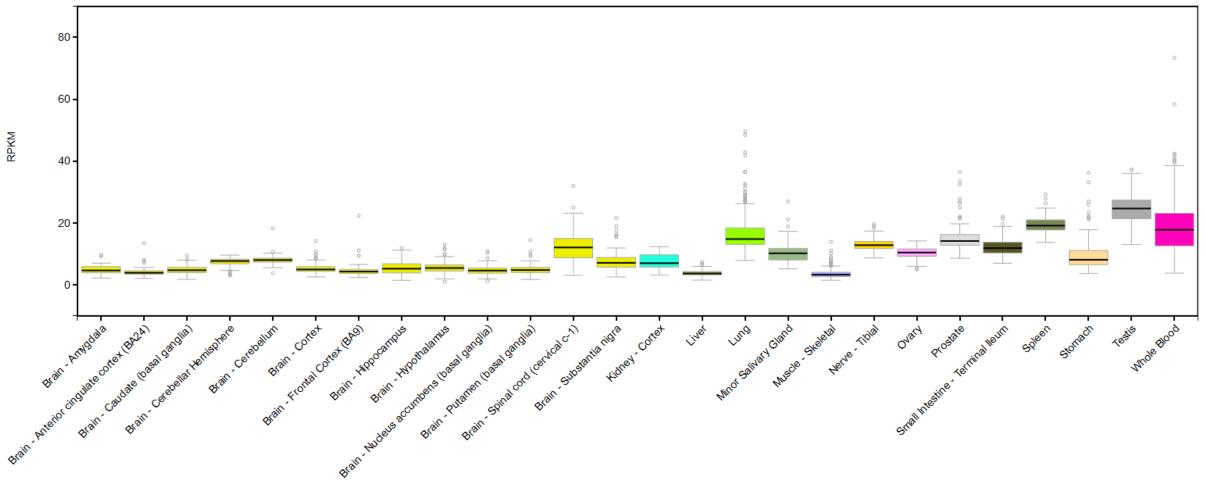

SFigure 4. TRAFD1 expression across different tissues in GTEx

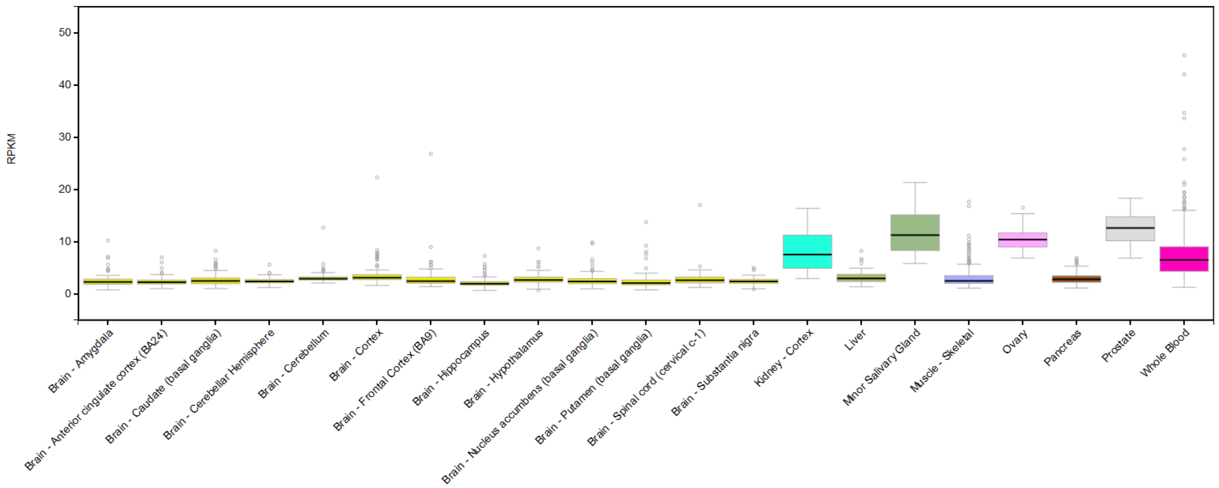

SFigure 5. PML expression across different tissues in GTEx
